# A deep learning approach to automatic detection of early glaucoma from visual fields

**DOI:** 10.1371/journal.pone.0206081

**Published:** 2018-11-28

**Authors:** Şerife Seda Kucur, Gábor Holló, Raphael Sznitman

**Affiliations:** 1 ARTORG Center for Biomedical Engineering Research, University of Bern, Bern, Switzerland; 2 Department of Ophthalmology, Semmelweis University, Budapest, Hungary; University of South Carolina, UNITED STATES

## Abstract

**Purpose:**

To investigate the suitability of multi-scale spatial information in 30^o^ visual fields (VF), computed from a Convolutional Neural Network (CNN) classifier, for early-glaucoma vs. control discrimination.

**Method:**

Two data sets of VFs acquired with the OCTOPUS 101 G1 program and the Humphrey Field Analyzer 24–2 pattern were subdivided into control and early-glaucomatous groups, and converted into a new image using a novel voronoi representation to train a custom-designed CNN so to discriminate between control and early-glaucomatous eyes. Saliency maps that highlight what regions of the VF are contributing maximally to the classification decision were computed to provide classification justification. Model fitting was cross-validated and average precision (AP) score performances were computed for our method, Mean Defect (MD), square-root of Loss Variance (sLV), their combination (MD+sLV), and a Neural Network (NN) that does not use convolutional features.

**Results:**

CNN achieved the best AP score (0.874±0.095) across all test folds for one data set compared to others (MD = 0.869±0.064, sLV = 0.775±0.137, MD+sLV = 0.839±0.085, NN = 0.843±0.089) and the third best AP score (0.986 ±0.019) on the other one with slight difference from the other methods (MD = 0.986±0.023, sLV = 0.992±0.016, MD+sLV = 0.987±0.017, NN = 0.985±0.017). In general, CNN consistently led to high AP across different data sets. Qualitatively, computed saliency maps appeared to provide clinically relevant information on the CNN decision for individual VFs.

**Conclusion:**

The proposed CNN offers high classification performance for the discrimination of control and early-glaucoma VFs when compared with standard clinical decision measures. The CNN classification, aided by saliency visualization, may support clinicians in the automatic discrimination of early-glaucomatous and normal VFs.

## Introduction

**Fig 1:**
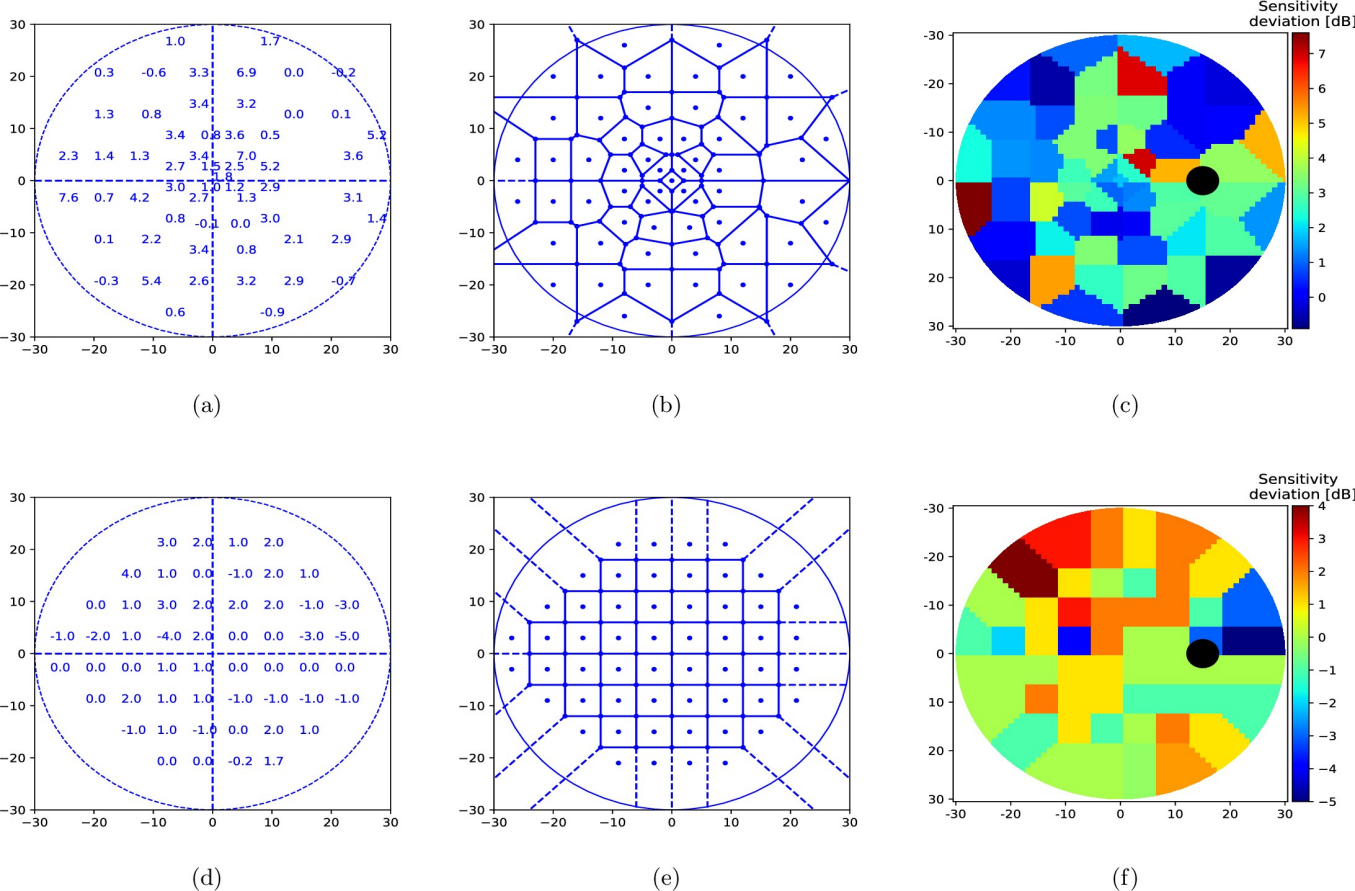
Voronoi image representation of 30° visual field. (a, d) Example OCTOPUS 101 perimeter G1 program and Humphrey Field Analyzer (HFA) 24–2 test patterns of 30°-visual field with age-normalized sensitivity thresholds, i.e. sensitivity deviation shown (b, e) Parcelation of the 30° visual field into a 61 by 61 pixel image using our proposed voronoi method. (c, f) Color-coded pixel values of computed voronoi image where cold colors (blue) depict high defect and warm colors (red) show low defect values.

Glaucoma is one of the most common, irreversible and potentially blinding progressive optic neuropathies, which is characterized with typical structural damage and functional deterioration [1,2]. Standard Automated Perimetry (SAP) is an essential tool in the diagnosis of glaucoma [3]. Perimetry provides quantitative evaluation on both the central visual field (VF) and the 24 to 30-degree visual field area via testing the predefined retinal locations for the individual *threshold sensitivity values*. The threshold sensitivity at a specific retinal location is the threshold light intensity (in dB) for which a subject can see a light stimulus with 50% likelihood. In general, the absolute threshold sensitivities are compared to age-related normal test point reference values in order to reflect *sensitivity deviations* within VFs. (see Fig 1A and 1D)). Sensitivity deviations provide the basis of the detection of glaucomatous VF defects.

Considering the progressive and irreversible nature of glaucoma, early diagnosis is of great clinical importance. However, glaucomatous VF deterioration first appears as small, localized relative defects. This makes obtaining the correct diagnosis challenging in early glaucoma. Since perimetry is a subjective test with several patient- and eye-related factors (*e*.*g*. measurement noise, poor signal-to-noise ratio, fatigue, fixation losses and learning effects), it is a limited assessment tool that can cause incorrect interpretation and classification in glaucoma [4]. To reduce the influence of noise on the classification result, conventional VF *global indices (e*.*g*. mean defect (MD), mean deviation, loss variance (LV), square root of loss variance (sLV), pattern standard deviation) are typically considered jointly for clinical classification. However, such indices do not reflect spatial information and may potentially fail to assist the detection of small and localized defects. Therefore, to optimize the detection of true early glaucomatous VFs, in clinical practice both the individual retinal sensitivity values and their locations in the VF, and the global indices need to be considered subjectively by clinicians. This process can be challenging for many ophthalmologists, in particular when glaucoma is at an early stage. An automated screening software that is capable of processing both the global and local information within the VF to discriminate patient-related noise from true glaucomatous functional alterations would provide significant support for the diagnostic process of glaucoma.

Several strategies for automated glaucoma detection from VFs have been proposed [5–8]. In combination with different Machine Learning classifiers (*i*.*e*. Artificial Neural Networks, K-Nearest Neighbors, Support Vector Machines), the use of individual threshold values or clinical indicators such as MD or sLV, have shown promising performances for discriminating healthy and early glaucomatous VFs [9]. Yet, these attempts fail to leverage spatial information within VFs, even though spatial relations have long been known to be useful for both glaucoma diagnosis [10] and defect pattern discovery [11]. Incorporating spatial information in a machine learning classifier may thus result in improved discrimination capabilities.

One classifier that explicitly takes into account spatial information at multiple sizes is the deep learning method of Convolutional Neural Network (CNN) [12], a form of Artificial Neural Network that uses spatial convolutions as the basis of its discrimination. While CNNs have been shown to be extremely effective in detecting biomarkers in OCT imaging [13] and for Diabetic Retinopathy grading [14], their use with VFs remains largely unexplored. This is mainly due to two reasons: (i) CNNs operate on images, whereby pixels have connected neighborhoods that can be defined at multiple scales. This quality allows CNNs to use local averaging and convolutions to extract features for discrimination tasks. However, VFs acquired from perimeters are not images and hence using CNNs on them is not possible in an unmodified manner. (ii) Beyond the ability to categorize VFs into two clinical groups, it is essential to determine which regions in the VF contain important information for decision-making. Hence, a strategy that visualizes which VF locations contribute significantly in a decision would meaningfully reveal and reduce the “black-box” nature of most automated strategies.

In this study, we present a novel method to discriminate between healthy and early-glaucomatous VFs using CNNs with an automated methodology. We introduce the concept of “voronoi images”, a method by which VFs acquired from a perimeter can be transformed into 2D images, regardless of the spatial distribution of the test locations within the 30-degree visual field. These voronoi images then allow us to use a custom designed CNN to classify VFs. Based on “saliency map” estimation [15,16], we provide two additional but different types of importance maps that highlight which VF regions contribute to the CNN decision. These maps are estimated directly from the CNN and are specific to each individual VF examination. In a next step, the effectiveness of our method was evaluated using two datasets of control and early-glaucomatous VFs and a comparison of our method with those based on MD, sLV and both, and a Neural Network (NN) that does not use spatial information was investigated.

## Method

We begin by describing our strategy to represent VFs as images and how these can then be used as inputs to a custom designed CNN. We then describe our method to highlight what regions of the VF are important to the CNN when classifying a VF.

### VF to image representation

VFs are converted to images using a voronoi parcelation [17,18] (Fig 1) that we name voronoi images. Each VF is divided in as many regions as the number of tested VF locations and each region is assigned the value at the corresponding tested location. To do so, we organize the raw data in a *L*-dimensional vector of sensitivity deviations *r*. Note that each element of *r*_*l*_,*l* = 1,2,…,*L* which we call seed points, correspond to a sensitivity deviation value at (*x*_*l*_,*y*_*l*_) coordinate location in the VF. Each seed point *r*_*l*_ is associated with its own region *R*_*l*_, such that any non-seed location is assigned to the region associated to the seed location to which it is closest. The resulting voronoi image can be formalized as a two-dimensional matrix *V*(*i*,*j*) such that

$$V(i,j)=r_{l^{*}},$$ (1)

where

$${l^{*}=argmin}_{l=1,2,\ldots ,L}{\left||\left(x_{l},y_{l}),(x,y\right)|\right|}^{2},\;0{}^{\circ}\leq x,y\leq 30{}^{\circ}.$$ (2)

With this representation, we can consider the VF as a two-dimensional image. An advantage of such an image representation is that it provides a way to represent perimetric data in a standardized form, regardless of the pattern used. Fig 1 illustrates converted VFs for two different test patterns (Fig 1A and 1D) used in clinics (Fig 1C and 1F)). Even though, the spatial distributions of the voronoi regions differ in the test patterns, they both share the same 2D image plane, and are therefore comparable to each other.

We treat each angle within a VF as a single pixel, such that the 30° tested area by the used test pattern corresponds to a 61 by 61 pixel image. Hence the VF origin (0°, 0°) is situated at image coordinate (31, 31). Note that left eye VFs are flipped to right eye configurations so to maintain a single orientation.

### Convolutional neural network classification

**Fig 2:**
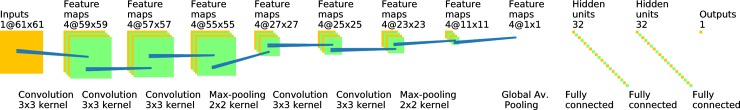
Proposed Convolutional Neural Network (CNN). The CNN takes as input a voronoi images (far left yellow square). Each stage corresponds to a layer in the network. The type of the layer and the filter sizes are described below each layer whereas the number of filters and the size of the output are given above each layer. Feature maps are finally averaged using a Global Average Max-Pooling layer. The final output score is obtained through two fully connected layers. All convolutional layers and the first two fully connected layer are followed by ReLU activation. The output layer has sigmoid activation and output score represents the probability of assigning the input visual field to the early glaucoma class.

We make use of an automatic and CNN-generated classification of a VF as control or EG. Our CNN takes as input a single voronoi representation of a VF and outputs 0 if the VF is from the control group and 1 if it is from the EG group. The CNN structure consists of 7 layers (see Fig 2), formed by 3x3 convolutional layers and batch normalization layers. Additionally, max-pooling layers perform down scaling by factors two on the output of each convolutional layer. A final binary decision is then produced using three fully connected layers to compute the VF output score (*i*.*e*. the probability of being early glaucomatous). Note that by using a CNN that explicitly uses convolution layers, we avoid the need to pre-smooth the voronoi representation of VFs, as such smoothing is inherent to the CNN. In particular, the CNN will learn features that are needed to achieve the defined classification task.

### Saliency maps

Two types of saliency maps are computed to visualize what region of a VF is contributing to its classification by the CNN. As proposed in [15], important pixels that contribute most to the output score of a convolutional neural network can be found by computing the gradient of the output with respect to the given input image. Let *O*_*c*_(*I*) be the output score associated with the class *c* and *VI* be the input voronoi image. Then, we compute a saliency map *S*_*C*_(*VI*) for the input image *VI* by differentiating *O*_*c*_ with respect to the image *I* as in the following:

$$S_{c}(VI)=\frac{\partial O_{c}(VI)}{\partial VI},$$ (3)

where ∂*y*/∂*x* refers to the gradient operator. The resulting *gradient image* reflects how much a small change in a pixel affects the output score. Therefore, such gradient image is expected to highlight important pixels or regions that bear relevant information with respect to the recognition task. However, as discussed in [16], such gradient images are in general noisy, which may be hard to interpret. Accordingly, we average the gradient images obtained by minimally perturbed input images (*e*.*g*. by adding Gaussian noise to the input image). We thus compute saliency maps as follows:

$${\hat{S}}_{c}(VI)=\frac{1}{n}\sum_{1}^{n}S_{c}(VI+N(0,\sigma^{2})),$$

where *n* is the number of samples to average over, *N*(0,*σ*^2^) is Gaussian noise with zero mean and standard deviation of *σ*. We refer to this map as *SmoothGrad*.

As our VF images are voronoi diagrams that are piece-wise constant, we further compute the mean of the SmoothGrad saliency maps within a voronoi image region and create a different map denoted *Piece-wise* computed by

$$S_{c}^{P}(x,y)=\frac{1}{n_{R_{l}}}\sum_{(x,y)\in R_{l}}{\hat{S}}_{c}(VI)(x,y)\;\forall l,\;l=1,2,\ldots ,L$$ (4)

where *R*_*l*_ is the voronoi region corresponding to the location *l*, $n_{R_{l}}$ is the number of pixels in the *R*_*l*_ and *L* is the total number of locations in the pattern. ${\hat{S}}_{c}(x,y)$ and $S_{c}^{P}(x,y)$ represent the (*x*,*y*) coordinates in the SmoothGrad and Piece-wise saliency maps, respectively.

Both SmoothGrad and Piece-wise maps are the same size as the voronoi image of a VF and normalized so that the range of values is between 0 and 1 (0 = no influence, 1 = maximal influence in the VF).

## Experimental setup

### Data

Two perimetry data sets were used in this study. VFs of the first data set were prospectively collected over a period of 10 years at the Glaucoma Center of Semmelweis University (Budapest, Hungary) using an OCTOPUS 101 perimeter (Haag-Streit AG, Köniz, Switzerland) with the G1 program test pattern (Fig 1A) and the normal test strategy. 3110 VFs were acquired at 6-month intervals from a mixed population comprising 107 eyes (healthy, ocular hypertensive [OHT], pre-perimetric and perimetric primary open-angle glaucoma eyes). The healthy eyes had no optic nerve head damage and had reliable and reproducible normal OCTOPUS G1 VF results, an MD < 2.0 dB, an LV < 6.0 dB, with no significantly decreased test point sensitivity values and intraocular pressure consistently below 21 mm Hg. The under treatment OHT eyes had normal optic nerve head and a normal VF with MD < 2.0 dB and LV < 6.0 dB. The under treatment perimetric glaucoma eyes had definite glaucomatous neuroretinal rim loss, and reliable and reproducible VF defects typical with glaucoma (inferior and/or superior paracentral or arcuate scotoma, nasal step, hemifield defect or generalized depression with MD > 2.0 dB and LV > 6.0 dB). Last, the under treatment preperimetric glaucoma eyes had glaucomatous neuroretinal rim loss reliable and reproducible normal OCTOPUS G1 VF results, an MD < 2.0 dB, an LV < 6.0 dB. In the current analysis, to neutralize the age-related differences between the eyes, the local defect values were used instead of absolute sensitivity thresholds. The VFs used in the current study were collected during a prospective clinical investigation, for which the research protocol was approved by the Institutional Review Board for Human Research of Semmelweis University, Budapest. Written informed consent was obtained from all participants before enrolment. All applicable institutional and governmental regulations concerning the ethical use of human volunteers were followed. All participants were white Europeans participating in a long-term imaging study in the Glaucoma Center of Semmelweis University in Budapest. We refer this data set as BD data set.

The second dataset used in this study was collected at the Rotterdam Eye Hospital [19,20] using a Humphrey Visual Filed Analyzer II (HFA, Carl Zeiss Meditec AG, Jena, Germany). Both eyes from 161 patients, 139 of whom are glaucomatous, were tested using a white-on-white 24–2 test pattern (Fig 1D), with the full-threshold program over 5 to 10 years, leading to 5108 visual fields in total. The diagnosis for each patient is provided within the dataset and the diagnosis criteria is described in [19,20]. Total deviation and MD values per visual field are also included in the data set which we refer to as RT.

The VFs used in the current investigation are categorized into two groups: control and Early Glaucomatous (EG). The control group comprises VFs of the normal, ocular hypertensive and pre-perimetric glaucoma eyes, all with MD < 6.0. The demographic statistics of the control and EG groups, along with MD and sLV means and range values are given in Table 1 and Table 2 for BD and RT data sets, respectively.

**Table 1:** Demographics of BD data set.

Group	# Eyes	# Visual Fields	MD (all VFs, mean; min; max)	sLV (all VFs, mean; min; max)
Control	114	1735	-0.31, -7.60;5.90	1.84; 0.90; 9.40
Early Glaucoma (EG)	87	532	3.16; -1.30;6.00 -	4.14; 1.10;12.60

**Table 2:** Demographics of RT data set.

Group	# Eyes	# Visual Fields	MD (all VFs, mean; min; max)	sLV (all VFs, mean; min; max)
Control	44	244	-0.05, -3.20;5.74	1.79; 1.05; 6.16
Early Glaucoma (EG)	220	2279	2.31; -5.12;6.00 -	3.74; 0.91;12.30

### Algorithm specifications

In this study, we use a 8-layer CNN model as seen in Fig 2, including 5 convolutional layers, 2 max-pooling layers, 1 global average pooling layer and 3 dense layers. Each convolutional layer applies 4 different 3x3 filters on its input, followed by a Rectified Linear Unit (ReLU) activation. Global average pooling is used after the last pooling layer, which outputs the average of each feature map. Two fully connected layers with 32 hidden units is added after global average pooling layer with an additional dropout layer with dropout factor of 0.5. Batch normalization with a batch size of 32 is applied after each layer except the last hidden layer where we apply dropout. Finally, we implemented an output layer of 1 hidden unit with sigmoid activation function which yields the class probability (*i*.*e*. the probability of being early glaucomatous).

Using a training set of VF and disease classification pairs, the CNN parameters are computed with the Adam optimizer [21] and the binary cross-entropy loss function. To study the performance and variance of the method, we split all VFs into 10 random subsets, and train 10 separate CNNs. Each CNN is trained with 9 unique subsets and validated on the remaining subset (*i*.*e*. 10-fold cross validation), making sure that no VF of a given subject appears in both the training and validations set.

When creating SmoothGrad saliency maps, we used the number of samples *n* = 500 and noise standard deviation *σ* = *σ*_*r*_ * (*VI*_*max*_−*VI*_*min*_) where *σ*_*r*_ is the noise level ratio, *VI*_*max*_ and *VI*_*min*_ are the maximum and minimum values in the voronoi image *VI* respectively. We accordingly used *σ*_*r*_ = 0.05 in our experiments.

### Prediction accuracy

Accuracy of the novel CNN was compared to conventional classification by MD and sLV. More specifically, MD is the negative of the arithmetic mean of sensitivity deviations, i.e.

$$MD=-\bar{r_{l}}$$ (5)

where $\bar{r_{l}}=\frac{1}{L}\sum_{l=1}^{L}r_{l}$, and sLV is square-root Loss Variance, i.e. the standard deviation of sensitivity deviations as given by

$$sLV=\sqrt{\frac{1}{L-1}\sum_{l=1}^{L}{{(r}_{l}-\bar{r_{l}})}^{2}.}$$ (6)

High MD and sLV values are often used as indicators of glaucoma and are complementary information to each other regarding visual function. We therefore evaluated them separately and combined as well, which we denote MD+sLV.

In addition, we compare these methods to a NN that has 2 fully connected hidden layers as in the top layers of our proposed CNN. NN uses the threshold values with global indices, i.e. MD and sLV as input to predict the VF group [8]. The predicted accuracy of each method was evaluated using the Average Precision (AP) defined by,

$$AP=\int_{0}^{1}{PPV}_{i}*{TPR}_{i}\;di,$$ (7)

where ${PPV}_{i}=\frac{\#true\;positives}{\#true\;positives+\#false\;positives}$ and ${TPR}_{i}=\frac{\#true\;positives}{\#true\;positives+\#false\;negatives}$ when applying a classification threshold 0≤*i*≤1 both. As such, a perfect score is achieved when AP = 1. In order to account for the variance due to neural network initializations, we trained our neural networks (CNN, NN) 5 times for each fold and computed the AP score for each single trained CNN/NN. The computed AP scores are then cumulated to calculate median and standard deviation values.

### Software

Data preparation, voronoi images, the CNN, saliency maps and analysis were performed using Python (publically available at https://www.python.org/). In addition, the Keras [22] deep learning software library (publically available at https://keras.io/) was used for the CNN implementation.

## Results

CNN and NN parameters were optimized during the training phase and a subset of training set was used as a validation set to avoid over-fitting of the two neural networks. The model that reached the best validation loss was selected to evaluate our approach on a separate test set. Computing a VF category and the two saliency maps with our CNN maps takes less than 0.1 second.

**Fig 3:**
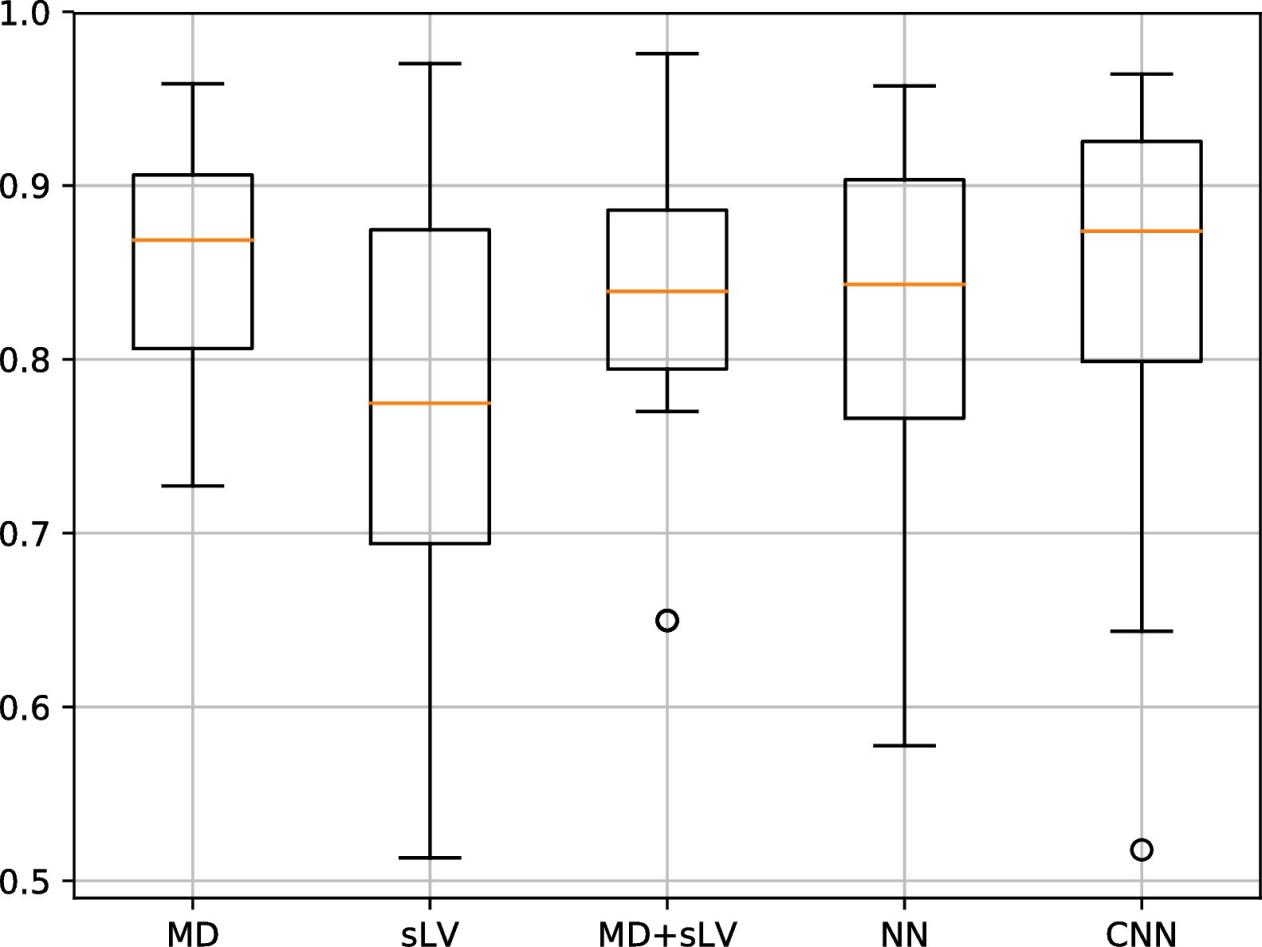
Average precision (AP) discrimination performance on BD data set. AP performance boxplots for MD, sLV, sLV+MD, NN and our CNN approach. Median values are shown in orange.

**Fig 4:**
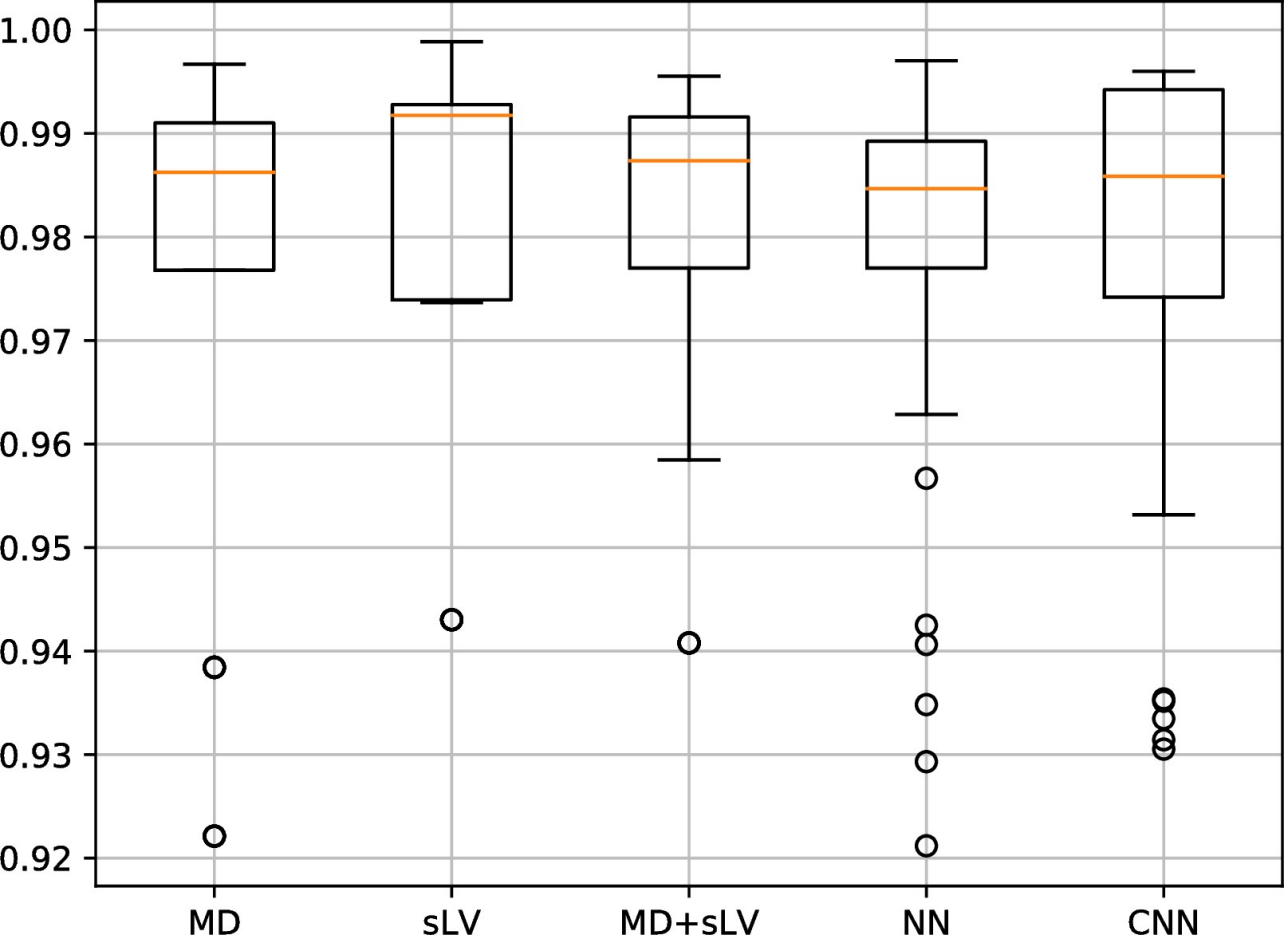
Average precision (AP) discrimination performance on RT data set. AP performance boxplots for MD, sLV, sLV+MD, NN and our CNN approach. Median values are shown in orange.

Figs 3 and 4 show the distribution of AP results associated with our CNN approach and that of MD, sLV, MD+sLV and NN for the BD and RT data sets, respectively. For the BD data set, the median AP scores over the 10 folds are 0.869±0.064, 0.775±0.137, 0.839±0.085, 0.843±0.089 and 0.874±0.095 for MD, sLV, MD+sLV, NN and CNN, respectively. For the RT data set, median AP scores are 0.986±0.023, 0.992±0.016, 0.987±0,017, 0.985±0.017 and 0.986±0.019 for MD, sLV, MD+sLV, NN and CNN, respectively.

**Fig 5:**
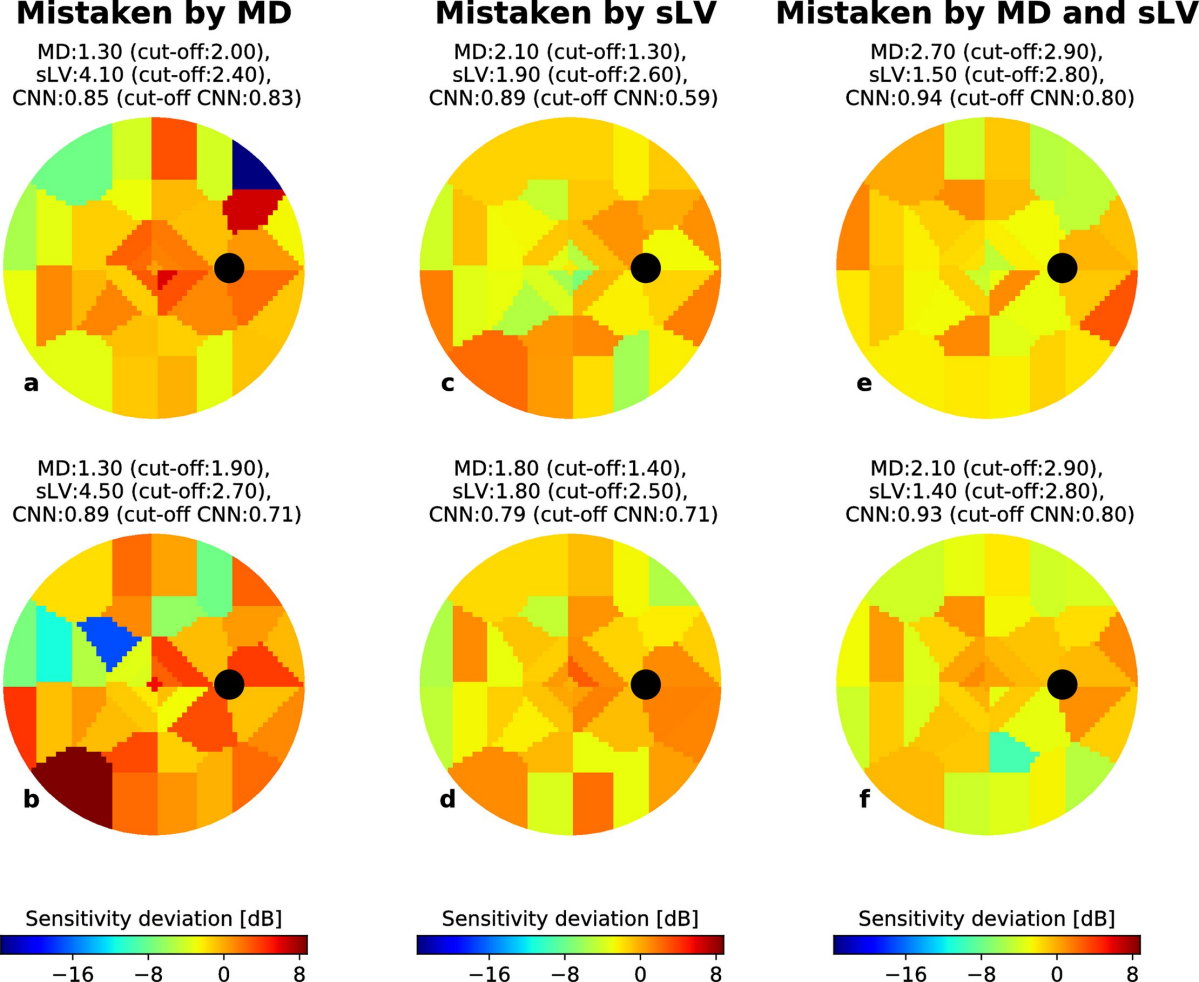
Correctly CNN-identified early glaucoma examples from the BD data set. For each example (a-f), the MD and sLV values are given above the voronoi visual field images. Black circles depict the blind spot. The probability of being early-glaucoma, estimated by the CNN is also given for each case. (a-b) Examples where an MD cut-off would lead to incorrect classification (cut-off values shown above each case). (c-d) Examples where an sLV cut-off would lead to incorrect classification (cut-off values shown above each case). (e-f) Examples where an MD+sLV cut-off would lead to incorrect classification (cut-off values shown above each case). In each case, the CNN detects the correct classification.

**Fig 6:**
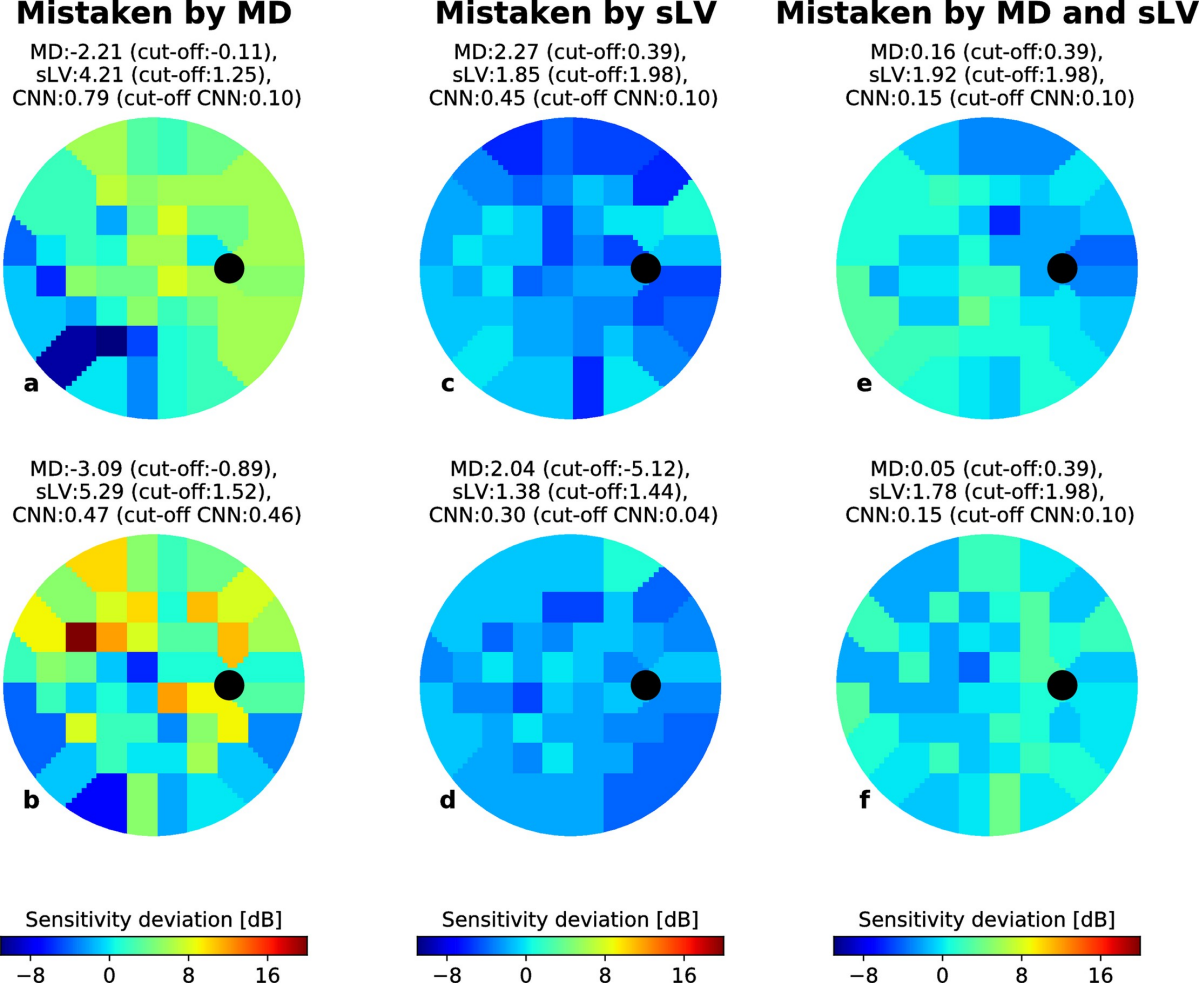
Correctly CNN-identified early glaucoma examples from the RT data set. For each example (a-f), the MD and sLV values are given above the voronoi visual field images. Black circles depict the blind spot. The probability of being early-glaucoma, estimated by the CNN is also given for each case. (a-b) Examples where an MD cut-off would lead to incorrect classification (cut-off values shown above each case). (c-d) Examples where an sLV cut-off would lead to incorrect classification (cut-off values shown above each case). (e-f) Examples where an MD+sLV cut-off would lead to incorrect classification (cut-off values shown above each case). In each case, the CNN detects the correct classification.

Figs 5 and 6 show randomly selected EG VFs, represented as voronoi images, for which the CNN correctly predicted the VF group but where MD, sLV and MD+sLV made incorrect predictions (cut-off used the maximum value of F1-scores defined by *PPV*_*i*_ * *TPR*_*i*_/(*PPV*_*i*_ + *TPR*_*i*_) over all values of *i*) for BD and RT data sets, respectively. For each case, the MD, sLV and CNN output scores are reported above the VF.

**Fig 7:**
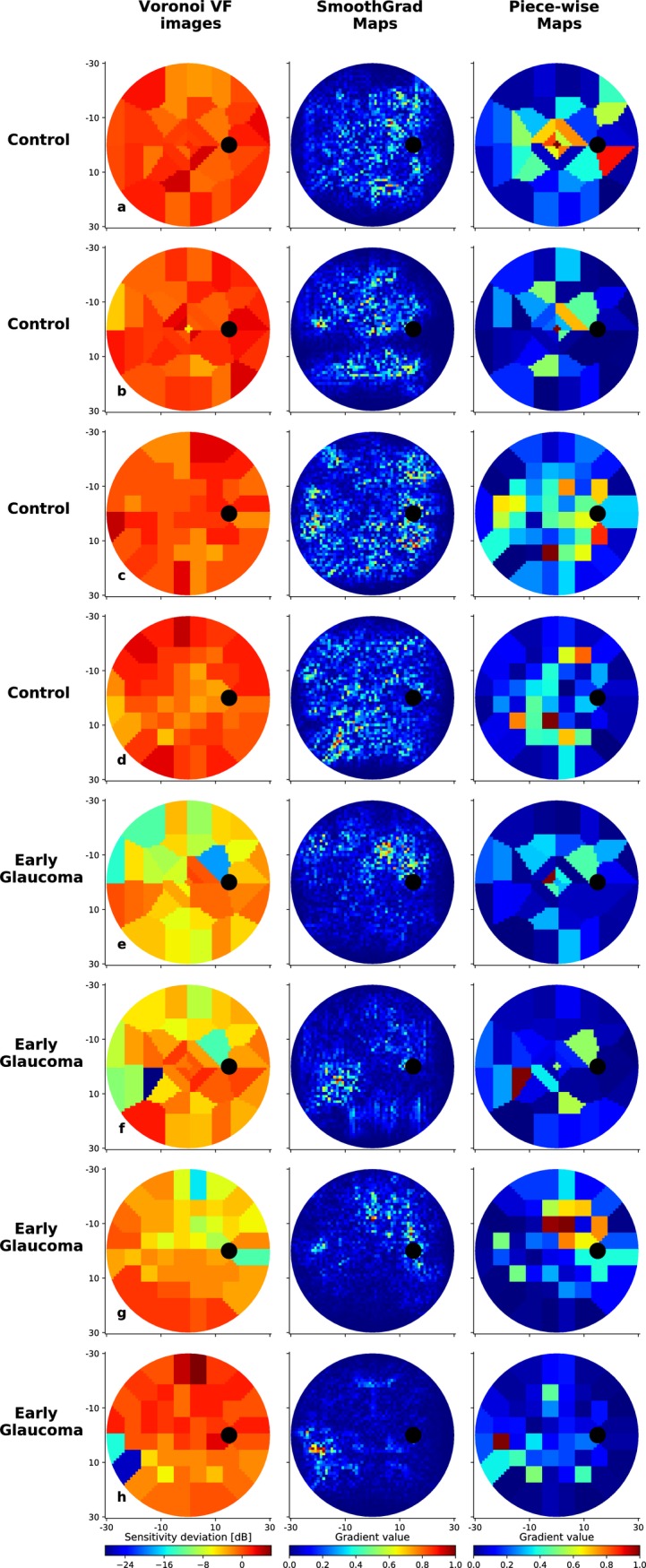
CNN-computed saliency maps. Eight visual fields correctly discriminated by the CNN: control cases from the BD data set with G program test pattern (a-b) and the RT data set with 24–2 test pattern (c-d); EG cases from the BD data set with G program test pattern (e-f) and the RT data set with 24–2 test pattern (g-h). For each case, both the SmoothGrad and Piece-wise saliency maps are shown. Values in the maps range from 0 to 1, where 0 indicates the pixel or region has no impact on the CNN decision while 1 indicates a region with maximal importance.

**Fig 8:**
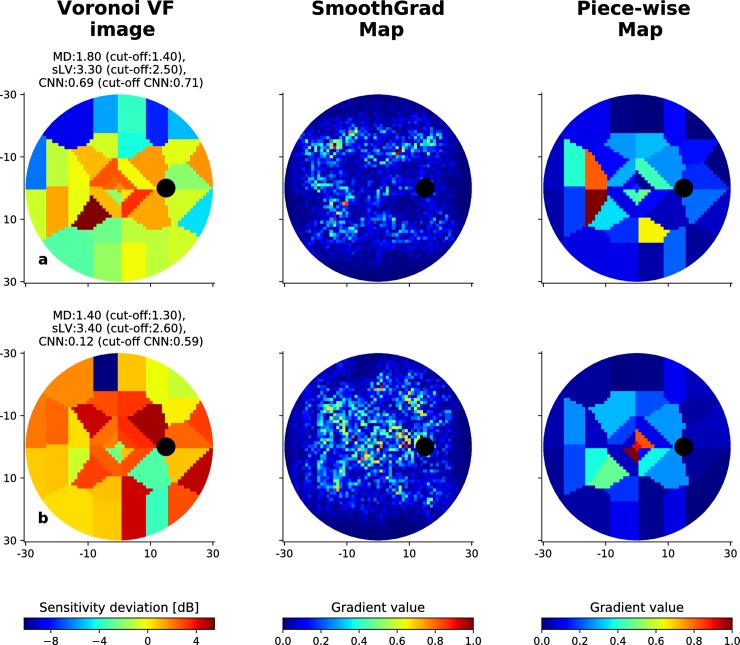
Incorrectly classified visual fields from the BD dataset. Illustration of two cases where the CNN fails to correctly identify early-glaucomatous visual fields, along with the associated SmoothGrad and Piece-wise saliency maps. MD and sLV values for each case are provided, as well as the probability of being early-glaucomatous estimated by the CNN. The corresponding cut-off values are given in parenthesis for each type of scores.

**Fig 9:**
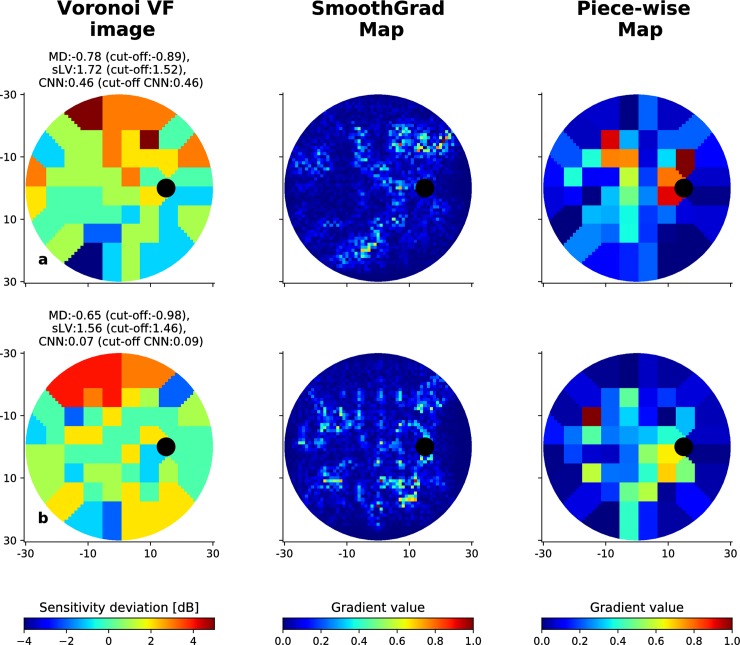
Incorrectly classified visual fields from the RT dataset. Illustration of two cases where the CNN fails to correctly identify early-glaucomatous visual fields, along with the associated SmoothGrad and Piece-wise saliency maps. MD and sLV values for each case are provided, as well as the probability of being early-glaucomatous estimated by the CNN. The corresponding cut-off values are given in parenthesis for each type of scores.

Fig 7 present randomly selected VFs and their associated SmoothGrad and Piece-wise saliency maps for BD and RT data sets. The red areas represent the regions that have greater influence on the CNN decision score than the other regions. Figs 8 and 9 show similar randomly selected examples for EG VFs, which are incorrectly indicated by the CNN as control.

## Discussion

In the current investigation we have shown that using a fully automated CNN to classify VFs into a control or an EG group via a voronoi image representation is more effective than using either a NN, MD, sLV and their combination for the same purpose.

As a general conclusion, the new approach investigated here, yielded consistently higher AP performances for the two data sets with different test patterns. sLV, which characterizes the homogeneity or inhomogeneity of the VF, and becomes impaired early in the glaucomatous progression, showed the worst performance and the highest variance in the BD data set while achieving highest AP performance on the RT data set, showing inconsistency in its performance across both data sets. This unexpected result may be explained by the fluctuations in patient responses[23]. However, the combination of MD with sLV improved the classification performance compared to that of MD alone. The improved performance of the CNN compared to that of the NN shows that spatial information over multiple region sizes results in higher discrimination capabilities, even if both neural networks use the same optimization method to train their respective parameters.

The variance achieved by the CNN method over the 10 folds was in general relatively large for both data sets whereas the difference was very small on the RT data set. This is due to some folds performing comparatively worse than the bulk of the folds with the CNN requiring a training set to establish the parameters of the model. This is borne out by work from others [14,24], and larger training data sets would reduce the variance of our CNN due to model parameters being less dependent on individual VFs.

The examples shown in Figs 5A, 5B, 6A and 6B suggest that the CNN is capable of detecting localized defects in VFs from different patterns. In Fig 5A and 5B, EG cases from the BD dataset were correctly identified by both sLV and the CNN (probability of early glaucoma is 0.85 and 0.89, respectively), but incorrectly classified with an MD cut-off of 2.00. In both cases, the sLV values are elevated (4.10 and 4.50, respectively) due to VF inhomogeneity. Similarly, EG cases from the RT data set, shown in Fig 6A and 6B, could be correctly classified by the CNN (probability 0.79 and 0.47) even though the EG samples are relatively harder with very low MD (-2.21 and -3.09) (Fig 5B). The low MD values suggest that this inhomogeneity has marginal influence on the global VF sensitivity, highlighting the challenge of detecting EG. However, these examples show that the CNN learned to associate EG to patterns that manifest as local inhomogeneity. At the same time, Figs 5A–5C, 6C and 6D highlight that the CNN also learned to take overall deviations into account. In the BD data set (Fig 5C and 5D), the CNN and MD cut-offs (1.30 and 1.40, respectively) lead to correct classification since the MDs are slightly higher than expected in normal control eyes (2.10 and 1.80, respectively). Here, VF inhomogeneity is negligible since the sLV scores are within normal values. We observe the same trend for the RT data set (Fig 6C and 6D) where the sLV did not suffice to identify EG cases with diffuse defects, while MD and CNN were successful. This suggests that the CNN is also able to identify EG cases that have diffuse defects with marginally elevated MD values. Figs 5E, 5F, 6E and 6F show more challenging cases where the CNN correctly classifies EG from both BD and RT data sets, while the combined MD+sLV with respective cut-offs leads to incorrect classification. Even when both the MD and sLV values are within normal ranges, the CNN correctly identified the EG cases.

Fig 7 highlights 8 different VF scores correctly classified by the CNN: two control from the BD data set (a-b), and the RT data set (c-d); two EG cases from the BD data set (e-f) and the RT data set (g-h). We note that the SmoothGrad maps contain significantly more noise than the Piece-wise maps. This is expected since the latter accumulates the values of the former over voronoi regions. In addition, the regions that are highlighted by the CNN (red regions) are not pre-set or constant for different VFs. That is, for each VF, the CNN used different combinations of locations to make the assessment.

For the control VFs in Fig 7A–7D, the SmoothGrad maps are often characterized by important regions that have large spatial coverage. More specifically, the control VFs appear to have SmoothGrad maps that are diffuse over the entire 30° of the VF, and attribute importance to many locations. Conversely, the maps associated with EG VFs are more spatially focused on few locations (Fig 7E–7H). One explanation for this difference is that for control VFs, the CNN needs to attribute importance to many locations so to verify that local defects are not present anywhere. In contrast, it is potentially enough to identify only a single defect region to classify the VF as early glaucoma. Of clinical significance is the finding that the regions highlighted in Fig 7E and 7H spatially correspond to the arcuate-like and nasal step paracentral scotoma areas, typical for early glaucoma[25,26].

For application purposes, the two different maps have their respective advantages: Piece-wise maps highlight the importance of each tested location by averaging SmoothGrad pixel influences over voronoi regions. However, this procedure may bias the importance of different Piece-wise regions because certain voronoi regions are larger than others. Conversely, SmoothGrad highlights each individual pixel in the voronoi image and suffers from noisy highlights. This suggests that considering both maps in combination is the most appropriate method to interpret the saliency map results.

The proposed CNN is not free from error. Figs 8 and 9 show two EG VFs that are incorrectly classified for the BD and RT data sets, respectively. Considering saliency maps associated to VFs from the BD data set in Fig 8, we observe that for the case in (a), defects on the upper hemi-field could be partially highlighted in the associated SmoothGrad map. In the case of Fig 8B, the CNN failed to focus on appropriate defect areas, thus resulted in much lower probability (0.12) than the cut-off value (0.59). As for cases in the RT data set shown in Fig 9A and 9B, SmoothGrad maps appear to highlight relevant regions that have low sensitivities whereas combinations of defect and non-defect locations were emphasized in Piece-wise map. Yet, the CNN could not accurately distinguish those VFs EG cases. This could be due to the complexity of the defect regions, where several isolated low-valued small regions are observed and are harder to correctly discriminate from inherent noise.

The study presented here has limitations and more extensive studies are required to confirm our initial findings, with future work focusing on two main areas. First, the need for large training cohorts for the CNN to learn a classification is essential for optimal performance. This would include training our method on extensive amounts of data arch. Using a greater amount of data, the complexity of the CNN (*i*.*e*. the number of layers of the model) can be increased, and concomitantly the accuracy will increase [27,28]. The second limitation is the current clinical criterion used to identify early-glaucoma subjects (*i*.*e*. considering only glaucomatous VFs having MD less than 6.0 dB). This criterion may in fact be incorrect. Our proposed CNN may provide a further improved detection of early glaucomatous VFs if it is trained using a further refined definition of EG.

In conclusion, in the current study we proposed a new CNN classifier to discriminate automatically between normal control VFs from EG VFs. We found that our approach performed better than the standard global indices used for clinical decision making. In addition, our proposed method outperformed a NN that does not explicitly leverage spatial information. We also emphasize that in such disease diagnosis problems, the need for interpretable results as opposed to single scores is critical and a main motivation for the introduction of the proposed saliency maps to highlight what regions of the VF the CNN focuses on during classification. The qualitative evaluation of these maps appears to correlate to regions that are clinically relevant. Our results represent a promising step for the integration of automated glaucoma detection methods in routine clinical practice and for research purposes. If developed further into a validated classification model, our proposed CNN method may gain a role in VF-based glaucoma screening in routine clinical care.

## Supporting information

S1 Dataset The is the file including data sets used in this work. Training, validation and test splits are given separately.
(ZIP)
